# Arrhythmia as the presenting symptom of a primary cardiac lymphoplasmacytic lymphoma

**DOI:** 10.1016/j.hrcr.2024.02.018

**Published:** 2024-03-05

**Authors:** Sumair Ozair, Gene Gerlach, Neha Patil, Karthik Venkatesh Prasad

**Affiliations:** North Mississippi Medical Center, Tupelo, Mississippi

**Keywords:** Lymphoplasmacytic lymphoma, Multimodality imaging, Atrial flutter, Primary cardiac tumors, Primary cardiac lymphoma

**Key Teaching Points**

• Lymphoplasmacytic lymphoma is a rare mature B-cell non-Hodgkin lymphoma primarily involving the bone marrow, liver, and spleen, with extranodal sites such as the skin and gastrointestinal tract. There have been limited case reports with cardiac manifestations.
• For patients with suspected primary cardiac lymphoma, electrocardiogram, echocardiogram, cardiac computed tomography (CT), cardiac magnetic resonance imaging, and positron emission tomography–CT should be completed to further characterize the tumor. Multimodality imaging is essential to further stratify the approach of care for a patient.
• When concerned for cardiac tumors, it is important to have a multidisciplinary approach, including cardiologists, cardiothoracic surgeons, and oncologists in the patient’s care.

## Introduction

Cardiac tumors are uncommon and are categorized into primary and secondary tumors. Secondary tumors are more common than primary tumors. Both types can present as lymphomas. Primary cardiac lymphomas (PCLs) account for around 1.3% of all primary cardiac tumors.^1^^,^^2^

Lymphoplasmacytic lymphoma (LPL) is a rare mature B-cell non-Hodgkin lymphoma primarily involving the bone marrow. Waldenstrom macroglobulinemia (WM) is a subtype of LPL characterized by IgM monoclonal gammopathy. The malignant cells in LPL are thought to originate from late stage of B-cell differentiation. Although not much is known about its etiology, LPL is associated with immunocompromised patients, often found in association with AIDS, hepatitis C, and autoimmune disorders. Additionally, there appears to be a familial predisposition—and those with this predisposition present at an earlier age.^3^^,^^4^

Typically, LPL is a diagnosis of exclusion, with all other small B-cell lymphomas ruled out first. Owing to its lack of specific morphologic, immunophenotypic, or chromosomal changes, diagnosing this form of lymphoma is challenging. Patients typically present with generalized B-related symptoms, such as fever, night sweats, and weight loss. Bone marrow involvement can cause fatigue and weakness related to cytopenias. Involvement of spleen, liver, and extranodal sites such as the skin and gastrointestinal tract have also presented as typical manifestations of this cancer.^3^^,^^5^

We report a case of primary cardiac LPL with a rather unusual presentation and its diagnostic and therapy approaches.

## Case report

**Figure 1:**
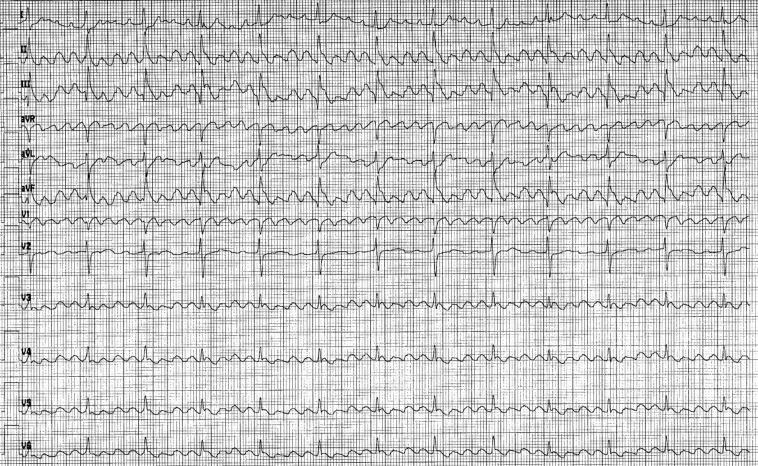
Electrocardiogram: Atrial flutter with variable block.

A 69-year-old woman with hypertension and class I obesity was evaluated by her primary care physician for chest discomfort and an electrocardiogram (ECG) was performed, showing atrial flutter (Figure 1). She was referred to a cardiologist, who started her on apixaban for anticoagulation for stroke prevention (based on her CHA_2_DS_2_-VASc score of 3) and initiated antiarrhythmic therapy with propafenone (class IC sodium channel blocker). She subsequently was referred to see a cardiac electrophysiologist and was recommended to undergo electrical cardioversion to restore sinus rhythm. The patient had no prior history of coronary artery disease, stroke, or congestive heart failure.

**Figure 2:**
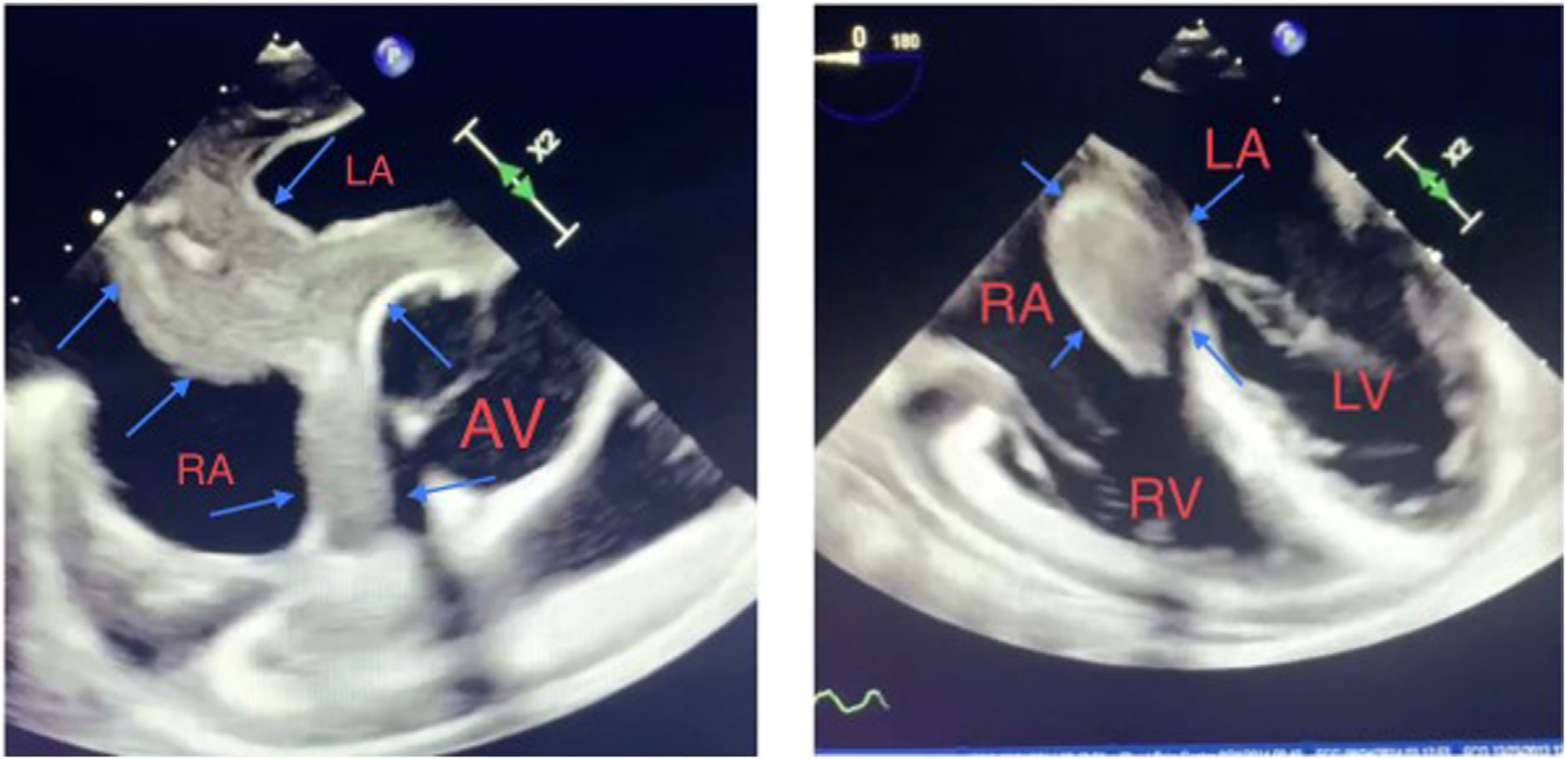
Transesophageal echocardiogram; blue arrows showing the abnormal mass involving the interatrial septum. AV = aortic valve; LA = left atrium; LV = left ventricle; RA = right atrium; RV = right ventricle.

**Figure 3:**
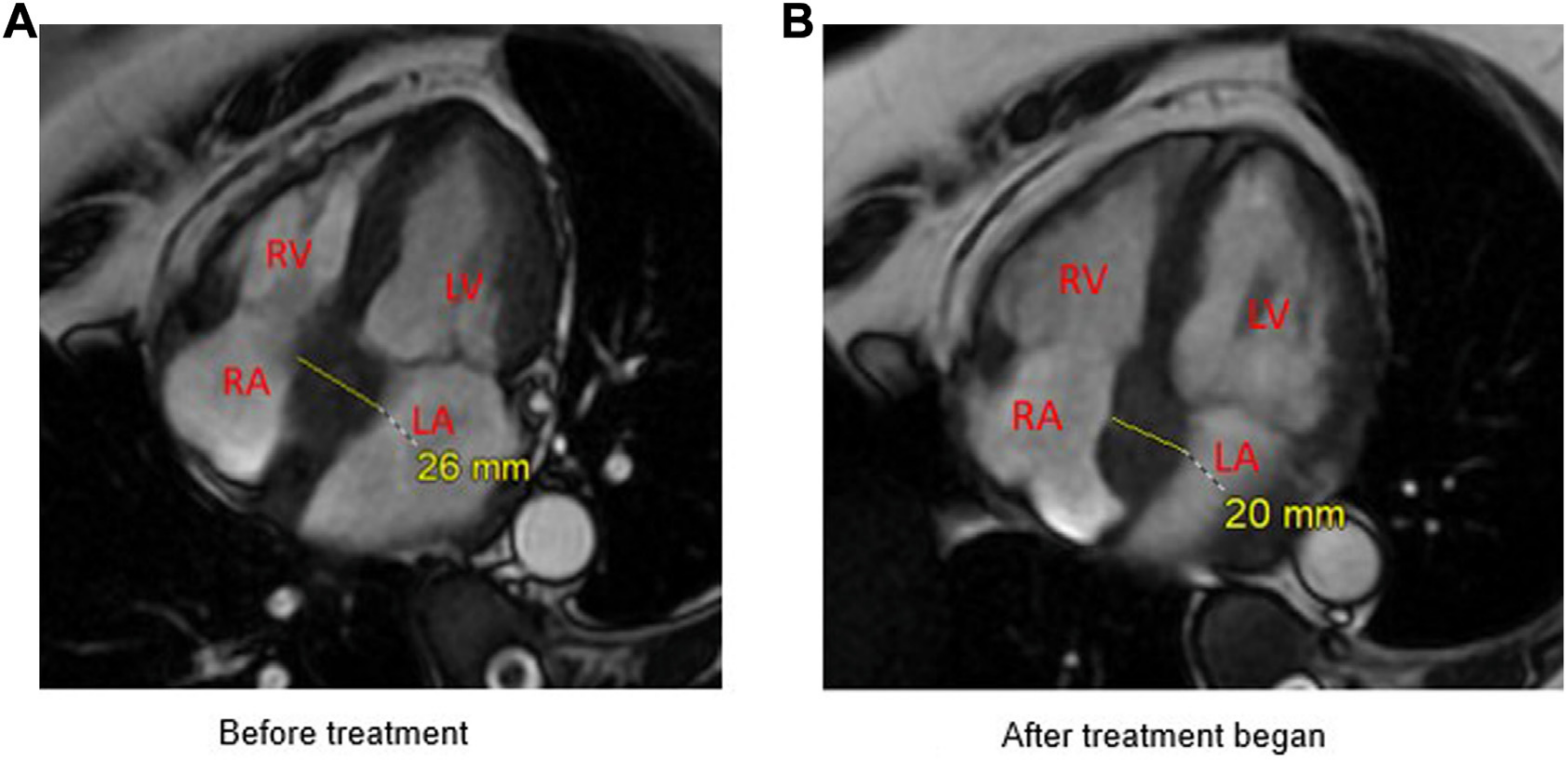
Cardiac magnetic resonance imaging; yellow lines denoting the size of the abnormal mass involving the interatrial septum. **A:** Before treatment. **B:** After treatment began. LA = left atrium; LV = left ventricle; RA = right atrium; RV = right ventricle.

Prior to the patient’s undergoing electrical cardioversion, a transesophageal echocardiogram (TEE) was performed to rule out left atrial appendage thrombus and evaluate for valvular heart disease. TEE was significant for abnormal thickening of the entire atrial septum and periaortic area measuring up to 2.8 cm with uniform echogenicity and smooth, well-defined borders. There was no thrombus in the right or left atria/appendages. Other findings were mild biatrial enlargement and mildly dilated left ventricle with a visually estimated ejection fraction of 50%–55% (Figure 2). Direct current cardioversion was performed with prompt restoration of sinus rhythm. With these abnormal TEE findings, further investigation was performed with cardiac magnetic resonance imaging (CMR) with delayed enhancement. CMR showed an infiltrative mass involving the interatrial septum that extends along the posterior left aortic valve groove and to a lesser extent superiorly around the posterior aspect of the aortic valve annulus (Figure 3A), with no late gas enhancement noted. Computed tomography (CT) scan of the chest, abdomen, and pelvis demonstrated mediastinal lymph node enlargement. Based on the above imaging tests, a preliminary diagnosis of extranodal lymphoma was made.

To get to a tissue diagnosis, percutaneous mediastinal lymph node biopsy was performed that demonstrated CD20+ B cells predominantly in the follicles and CD3+ T cells predominant elsewhere, along with kappa and lambda in situ hybridization with mild kappa skew. This was inconclusive for a lymphoma diagnosis. A slightly more invasive mediastinoscopy was performed for biopsy of paratracheal and right carinal lymph node. This demonstrated kappa light chain skewed plasma cells with CD20 stained B cells within follicles and paracortex, CD3 stained paracortical T cells, CD10 stained germinal centers, CD21 and CD23 stained intact follicular dendritic meshworks, and PD-1 stained T-follicular helper cells in the lymphoid follicles. These biopsy results were more characteristic but still not definitive of lymphoma.

With the above inconclusive results, a transvenous right atrial mass biopsy was performed which was definitive for lymphoma. Biopsy showed LPL, MyD 88 mutation positive, small B-cell lymphoma with plasmacytoid features negative, Congo red stain negative, and CD20 positive. Although extracardiac lymph nodes did demonstrate some characteristics of lymphoma, the cardiac biopsy was the only definitive sample.

Positron emission tomography/CT (PET/CT) scan showed mild activity within the left para-aortic mediastinal nodal mass, with uptake greater than the mediastinal blood pool but less than or equal to the liver. Deauville score was 3. Bone marrow biopsy with cytogenetics was overall normal.

A final diagnosis of primary cardiac LPL was made and the patient was subsequently referred to hematology-oncology. Being symptomatic with cardiac involvement, she was promptly started on systemic chemotherapy with bendamustine plus rituximab (BR).

CMR after cycle 3 of BR showed significant improvement in the mass (Figure 3B). No new lymphomatous involvement was seen, with no new lymphadenopathy. PET scan after cycle 6 showed complete remission, with previously noted periaortic lymph node showing no abnormal PET scan activity. Deauville score was 2. No new pathologically enlarged or metabolically active lymphadenopathy were seen.

She is currently on surveillance and had follow-up echocardiograms and CT chest abdomen pelvis scans, which were overall stable. More recent TEE demonstrated decrease in size and thickness of interatrial septum with significant decrease in size of mass. Patient atrial flutter and chest pain is much improved, with an overall improvement in her quality of life.

## Discussion

As previously discussed, LPL is a rare disease typically involving the bone marrow, lymph nodes, liver, and spleen. Even more rare is its presentation as a primary cardiac tumor (PCT). The majority (around 90%) of PCTs are benign. PCL is a subset of malignant PCTs, which comprise of 1.3% of all PCTs. PCLs invade the cardiac cavity and/or pericardium and are characterized by cardiac symptoms—primarily within the right atrium, followed by the right ventricle, the left ventricle, and, lastly, the interatrial septum. Cardiac involvement as initial presentation of lymphoma is uncommon, however patients can present with congestive heart failure, dyspnea, syncope, atrial or ventricular arrhythmia, pulmonary embolism, pericardial effusion, pleural effusion, myocardial infarction, stroke, and sudden cardiac death if immunocompromised.^1^, ^2^, ^3^^,^^6^

Given how rare PCLs are, upon literature review there were no data available regarding LPL specifically in the heart. Broadly speaking, PCLs are most commonly found to be diffuse large B-cell lymphoma (DLBCL) that involves the right atrium primarily. Eighty percent of PCLs in immunocompetent patients are diffuse large B-cell lymphoma.^3^^,^^6^

For patients with suspected PCL, ECG, echocardiogram, cardiac CT, CMR, and PET-CT should be completed. ECG findings are not specific to diagnose, and may demonstrate atrial flutter, atrial fibrillation, bundle branch block, and low voltage. Transthoracic echocardiography may help detect cardiac masses. Cardiac magnetic resonance imaging is the best imaging modality to further distinguish between the cardiac tumor and cardiac tissue with better imaging of cardiac structures. PET-CT is used to assess invasion and metabolic activity of the tumor by noting increased glucose metabolism.

True final diagnosis is dependent on biopsy with specific markers. On flow cytometry LPL cells are surface IgM+, CD19+, CD20+, CD22+, CD25+, CD27+, FMC7+, CD5 variable, CD10-, CD23-, CD103-, and CD108-. Plasma cells are typically CD138-. In order to assess paraprotein in LPL, electrophoresis and immunofixation are completed. There is no specific chromosomal abnormality for LPL; however, those patients with an MyD88 L265P mutation favor diagnosis. But this mutation is nonspecific and not necessary for diagnosis. In addition to the MyD88 L265P mutation (95% of patients), other mutations such as CXCR4 (30%–40%), ARID1A (17%), and CD79B (8%–15%) may allude to an LPL diagnosis. All described diagnostic testing was completed in this case to fully assess the patient’s clinical presentation.^7^, ^8^, ^9^

Treatment modality for symptomatic patients with LPL/WM typically includes rituximab-based therapy with systemic chemotherapy or oral Bruton tyrosine kinase inhibitors for older patients who do not want systemic chemotherapy. Typically, patients are started with BR regimen—the preferred first-line treatment for LPL/WM. Bendamustine induces apoptosis and inhibits cell division while rituximab is a monoclonal antibody that targets CD20 that triggers cell lysis of those with that antigen. Clinical trials have demonstrated high response rates with improved median progression-free survival and good tolerability with this regimen compared to other regimens. Stem cell transplant is typically reserved for younger patients with good performance status, those with relapsed or refractory disease, and those in whom other treatment options have been exhausted.^3^^,^^10^, ^11^, ^12^, ^13^, ^14^, ^15^

The paucity of data was one of the greatest limitations in this case. Extensive medical management from multiple subspecialities was necessary to accurately diagnose and treat this rare cancer in our patient. This single case report brings to light the importance of further research into primary cardiac LPL and the clinical implications of this diagnosis. By focusing more attention on this topic, we as a medical community can better understand the origin of these forms of cancers and their specific locations.

## Conclusion

This is a rare case of primary cardiac LPL that presented with minimal symptoms aside from nondescript chest pain and arrhythmia. It is hoped that this case broadens our understanding of LPL and how it may present.
